# Getting It Right the Second Time: How Can we Optimize First‐Generation Cephalosporin Dosing for Skin and Soft Tissue Infections in the 21st Century?

**DOI:** 10.1002/phar.70179

**Published:** 2026-06-10

**Authors:** Jonathan H. Ryder, Shawnalyn W. Sunagawa, Nathaniel J. Rhodes, Trevor C. Van Schooneveld, Jeremy E. Tigh, Sean N. Avedissian, Nicolas Cortes‐Penfield

**Affiliations:** ^1^ Department of Internal Medicine, Division of Infectious Diseases University of Nebraska Medical Center Omaha Nebraska USA; ^2^ Department of Pharmacy Practice and Science University of Nebraska Medical Center Omaha Nebraska USA; ^3^ Antiviral Pharmacology Laboratory, College of Pharmacy University of Nebraska Medical Center Omaha Nebraska USA; ^4^ Department of Pharmacy Practice Midwestern University Downers Grove Illinois USA; ^5^ Pharmacometrics Center of Excellence Midwestern University Downers Grove Illinois USA; ^6^ Department of Pharmacy Good Samaritan Regional Medical Center Corvallis Oregon USA

**Keywords:** cefadroxil, cephalexin, first‐generation cephalosporins, pharmacokinetics, skin and soft tissue infection

## Abstract

Optimal cephalexin and cefadroxil dosing for skin and soft tissue infections (SSTIs) is unclear. We summarize clinical and pharmacokinetic/pharmacodynamic (PK/PD) data that compare dosing strategies for SSTIs. Additionally, we conduct population PK target attainment simulations for varying doses of cephalexin and cefadroxil for *Staphylococcus aureus* and Group A *Streptococcus*. Although some clinical data support lower doses in mild SSTIs, higher doses optimize PK/PD parameters in moderate–severe SSTIs, especially due to *Staphylococcus aureus* . Further prospective clinical and PK/PD studies, especially in obese adults, would be beneficial.

## 1 Introduction

Treatment failure in skin and soft tissue infections (SSTIs) remains high, averaging 18% (range 6%–37%) across 20 clinical trials evaluating cellulitis [1]. This is likely multifactorial, including medical comorbidities (e.g., obesity, heart failure, diabetes, and chronic kidney disease), misdiagnosis of cellulitis mimickers, and the delayed improvement as part of the natural history of cellulitis [1, 2, 3]. However, inadequate pharmacokinetic (PK) target attainment and potential nonadherence to frequently‐dosed antibiotics for SSTI (e.g., cephalexin, clindamycin) could be other contributing factors. Nonadherence to antibiotic therapy in SSTI is unsurprisingly associated with inferior clinical response [4]. A systematic review of medication adherence found that across 76 studies, mean dose‐taking declined in a dose‐dependent manner from 79% with daily therapy to 51% with four times daily treatment [5]. Given these challenges and associated risk of treatment failure, regimens with less frequent dosing are attractive.

Oral first‐generation cephalosporins (e.g., cephalexin and cefadroxil) are commonly prescribed for SSTIs in the United States [6], but the optimal dosing of these antibiotics for SSTI is not well understood. The most common gram‐positive organisms responsible for SSTIs include beta‐hemolytic Streptococci (BHS), including Group A Streptococcus (GAS), and methicillin susceptible *Staphylococcus aureus* (MSSA), both of which are routinely susceptible to oral first‐generation cephalosporins [7]. The labeled dosing for oral cephalexin in adults is 250 mg every 6 h or 500 mg every 12 h [8], whereas the Infectious Diseases Society of America (IDSA) SSTI guidelines recommend a dose of 500 mg every 6 h (Table 1) [10]. For cefadroxil, labeled dosing is 1 g daily or 500 mg two times daily [9], whereas IDSA guidelines do not provide dosing guidance [9]. Adherence to the four times daily regimen of cephalexin is challenging, as exemplified by 18% of patients taking < 75% of cephalexin doses in a highly controlled trial setting, so less frequent regimens would be desirable [11]. Thus, clinicians currently lack clear guidance on optimal dosing of oral first‐generation cephalosporins in moderate–severe SSTI. Herein, we summarize available studies of cephalexin and cefadroxil, including PK and clinical outcomes, in adult patients with SSTI as well as conduct population PK target attainment simulations to evaluate the likely target attainment with contemporaneous dosing regimens.

**TABLE 1:** Comparison of cephalexin and cefadroxil recommended dosing.

Recommending organizations	Cephalexin		Cefadroxil	
FDA Approved Doses [8, 9]	250 mg four times daily 500 mg two times daily Severe infections: up to 4 g per day in 2–4 divided doses		1 g daily 500 mg two times daily	
FDA Recommended Dosing Regimens for Renal Impairment	CrCl (mL/min)	Recommended Dose	Initial dose of 1000 mg followed by the maintenance dose of:	
	≥ 60	No dose adjustment	CrCl (mL/min)	Recommended dose
	30–59	No dose adjustment; maximum dose of 1 g/day	≥ 50	No dose adjustment
	15–29	250 mg two or three times daily	25–50	500 mg two times daily
	5–14	250 mg daily	10–25	500 mg daily
	1–4; not yet on dialysis*	250 mg every 48 h or every 60 h	0–10	500 mg every 36 h
IDSA Guidelines Recommended Dose for SSTI [10]	500 mg four times daily		None	

Abbreviations: CrCl, creatinine clearance; FDA, Food and Drug Administration; IDSA, Infectious Diseases Society of America; SSTI, skin and soft tissue infection.

* Insufficient information to make recommendations for patients on hemodialysis.

## 2 Literature Search

We reviewed observational studies and randomized controlled trials (RCTs) listed in PubMed from inception through January 23, 2025 that compared dosing regimens for cephalexin and/or cefadroxil in adults with SSTIs, or that reviewed cephalexin and/or cefadroxil PK/pharmacodynamics (PD) with attention to the impact of dosing strategies and/or obesity. The full text query and search parameters are included in the Supporting Methods. Table 2 describes included studies, study design, inclusion/exclusion criteria, and relevant outcomes. Studies are described in the text categorically by differing designs (e.g., PK vs. clinical), unique patient population (e.g., obesity), and intervention (dose or frequency).

**TABLE 2:** Included clinical studies of adult patients with SSTI treated with varying doses and frequencies of cephalexin and cefadroxil.

Author (year)	Study design (sample size)	Included diagnoses/severity (*n*/%)	Key patient characteristics, *n* (%)	Key inclusion/exclusion criteria	Primary study outcome(s)	Drug and dose comparisons	Outcome, *n* (%)
High vs. low dose (same frequency)							
Johnson (1972) [12]	Sub‐group of RCT (*n* = 217)	Soft tissue infections (mild 7.4%; moderate 65.9%; severe 26.7%)	Age: NR for soft tissue infection sub‐group Sex: 53% male Weight: NR	Inclusion: age > 15 year Exclusion: Markedly impaired renal function	Satisfactory response: “recovered” by day 7, “marked improvement” on day 7 without further antibiotic treatment, or recovered by day 11 when treated beyond day 7	Cephalexin 250 mg four times daily vs. Cephalexin 500 mg four times daily	100/105 (95%) vs. 103/112 (92%)
Browning (1981) [13]	Sub‐group of RCT (*n* = 234)	Soft tissue infections including cellulitis, abscesses, boils, carbuncles (*n* = NR)	NR for soft tissue infection sub‐group	Inclusion: age > 15 year Exclusion: NR	Success (complete remission of signs/symptoms) or considerable improvement (signs/symptoms much improved)	Cephalexin 500 mg two times daily vs. Cephalexin 1 g two times daily	102/120 (85%) vs. 100/114 (88%)
Ballantyne (1982) [14]	“Open” study (*n* = 373)	SSTIs	NR	Inclusion: age ≥ 6 year Exclusion: hepatic/renal impairment, pregnancy	Clinically and bacteriologically satisfactory response	Cefadroxil 500 mg two times daily vs. Cefadroxil 1 g two times daily	157/158 (99%) vs. 200/215 (93%)
Ballantyne (1982) [14]	“Double‐blind comparative” study (*n* = 78)	Furunculosis	NR	Inclusion: age ≥ 6 year Exclusion: hepatic/renal impairment, pregnancy	Clinically and bacteriologically satisfactory response	Cefadroxil 500 mg two times daily vs. Cefadroxil 1 g two times daily vs. Cephalexin 500 mg two times daily vs. Cephadrine 500 two times daily	18/18 (100%) vs. 18/18 (100%) vs. 20/22 (91%) vs. 20/20 (100%)
Yadav (2023) [15]	Pilot RCT (*n* = 66)	Cellulitis	Age (median): 56–57 year Sex: 39.4% female Obesity: 45.5%	Inclusion: age ≥ 18 year in ED Exclusion: abscess, malignancy, febrile neutropenia, organ transplant, creatinine clearance < 30 mL/min	Oral antibiotic treatment failure (change in oral antibiotic class or escalation to intravenous antibiotics)	Cephalexin 500 mg four times daily vs. Cephalexin 1 g four times daily	4/31 (12.9%) vs. 1/31 (3.2%)
More vs. less frequent (same total daily dose)							
DiMattia (1981) [16]	RCT (*n* = 133)	SSTIs including impetigo (57.9%), cellulitis (14.3%), subcutaneous abscess (13.5%), and miscellaneous including postoperative wounds, eczema, ecthyma, and paronychia (14.3%)	Age (range): 1 month to > 70 year Sex: NR Weight: NR	Inclusion: NR Exclusion: foreign material, systemic infections, pregnancy	Satisfactory clinical outcome (resolution/improvement in signs/symptoms or initial improvement on therapy but later recurred) and bacteriologic cure (resolution of inflammation and clearance of pathogen or recurrence due to new pathogen)	Cephalexin 250 mg four times daily vs. Cephalexin 500 mg two times daily	65/65 (100%) vs. 66/68 (97%)
Ballantyne (1982) [14]	“Double‐blind” study (*n* = 180)	SSTI	NR	Inclusion: age ≥ 6 year Exclusion: hepatic/renal impairment, pregnancy	Clinically and bacteriologically satisfactory response	Cefadroxil 1 g daily vs. Cefadroxil 500 mg two times daily vs. Cephalexin 500 mg four times daily	59/60 (98%) vs. 57/59 (97%) vs. 60/61 (98%)
Increased frequency of same dose							
Cordero (1976) [17]	Observational (*n* = 36)	Bacterial SSTI including abscess (8.3%), cellulitis/erysipelas (13.8%), furunculosis (47.2%), hidradenitis (8.3%), carbuncles (8.3%), and *n* = 1 each of eczematoid pyodermitis, impetigo, pyodermitis, and ulcer	Age (range): 12–83 year Sex: 72.2% male Weight: NR	NR	Clinical cure (resolution of signs and symptoms) and bacteriologic cure (sterile post‐treatment cultures or healing of lesions if no post‐treatment cultures possible)	Cefadroxil 300 mg two times daily vs. Cefadroxil 300 mg three times daily	25/25 (100%) vs. 11/11 (100%)
Cefadroxil vs. cephalexin							
Ballantyne (1982) [14]	“Double‐blind” study (*n* = 180)	SSTI	NR	Inclusion: age ≥ 6 year Exclusion: hepatic/renal impairment, pregnancy	Clinically and bacteriologically satisfactory response	Cefadroxil 1 g daily vs. Cefadroxil 500 mg two times daily vs. Cephalexin 500 mg four times daily	59/60 (98%) vs. 57/59 (97%) vs. 60/61 (98%)
Gooch (1991) [18]	RCT (*n* = 223), also included a cefuroxime arm	SSTIs including furunculosis (30.4%), cellulitis (27.8%), impetigo (21.5%), folliculitis (8.5%), carbuncles (10.3%), injury‐induced wound (7.6%), and other (13.9%) 74% moderate severity	Age: mean 41–42 year with range 4–89 year Sex: 55%–60% male Weight (lbs): mean 161–170 with range 44–350	Inclusion: age ≥ 4 year, mild–moderate SSTIs Exclusion: pregnancy	Satisfactory clinical response, including cure (resolution of symptoms/healed lesion 1–3 days after treatment with no further treatment required) or improvement (resolution of symptoms but incomplete healing 1–3 days after treatment)	Cefadroxil 500 mg two times daily vs. Cephalexin 500 mg two times daily	82/87 (94.3%) vs. 80/90 (88.9%)

Abbreviations: ED, emergency department; NR, not reported; RCT, randomized controlled trial; SSTI, skin and soft tissue infection.

## 3 Definitions

We discuss and interpret relevant PK/PD and clinical data within the context of the severity of SSTI according to definitions adopted and modified from the IDSA SSTI guidelines [10]. Mild infections include generally outpatient SSTIs without systemic signs of infection and include superficial SSTIs that do not necessarily require systemic antibiotic therapy such as impetigo, folliculitis, furuncles, carbuncles, and small abscesses. Moderate SSTIs include outpatient and inpatient SSTIs with systemic signs of infection. Severe SSTIs are exclusively inpatient and may require extensive surgical debridement, have hemodynamic instability or require intensive care unit level of care, and may have concomitant bacteremia. The primary focus of this manuscript will be on moderate–severe infections, specifically when at a point in the clinical course where oral therapy is considered (e.g., after resolution of hemodynamic instability and initial debridement), whereas mild infections are of lower clinical concern because current dosing strategies are likely adequate for these infections or they may not require any systemic antibiotic therapy for successful treatment. Individual studies often used their own definitions for severity, which can lead to variability in interpretation.

## 4 Pharmacokinetic Simulation Methods

We evaluated published PK studies of cephalexin and cefadroxil to determine PK/PD target attainment with various oral dosing regimens [19]. Previously published pediatric population PK models, which primarily simulated higher doses, were employed to simulate adult cephalexin and cefadroxil target attainment via allometry [19, 20]. Adult simulations were further supplemented with oral PK anchors derived from published summary data using a pharmacometrics mixture approach [21, 22]. Monte Carlo simulations evaluated regimens against minimum inhibitory concentration (MIC) distributions using 15% protein binding, estimating probability of target attainment (PTA)/cumulative fraction of response (CFR) at 40% (primary) and 90% (conservative) free time above the MIC (*f*T_>MIC_). Additionally, a CFR analysis was also performed for each drug to evaluate target attainment against GAS and MSSA at PK/PD goals of 40% and 90%. MIC distributions for each organism used in the CFR analysis were obtained from the European Committee on Antimicrobial Susceptibility Testing when available (Supporting Methods) [23].

Specifically, we used the highest weight group represented in those models for each antibiotic (~70 kg) to extrapolate to adult dosing, which was captured in both the apparent volume of distribution (Vd) and clearance (CL) terms (i.e., CL/F = typical CL * [Weight/70]^0.75^, Vd/F = typical Vd * [Weight/70]^1^). We simulated cephalexin doses of 500 mg or 1000 mg orally every 6, 8, or 12 h. For cefadroxil, we simulated doses of 500 mg or 1000 mg orally every 8 or 12 h. We compared PK/PD target attainment versus MICs in doubling dilutions for GAS (0.06–0.5 mg/L) and MSSA (0.5–16 mg/L) with goals of 40% or 90% fT>MIC for each regimen [24]. For simulations and PK/PD target attainment, a 15% protein binding value was used for both cephalexin and cefadroxil, consistent with previous studies [19, 25, 26].

## 5 Pharmacokinetics and Pharmacodynamics of Cephalexin and Cefadroxil Review

Cephalexin and cefadroxil have favorable PK/PD parameters including high oral bioavailability, minimal hepatic metabolism, and low protein binding [8, 27, 28]. Studies directly comparing cephalexin and cefadroxil PK/PD are described in the Table S1. In a parallel group design comparing 250, 500, and 1000 mg of cefadroxil and cephalexin in 36 healthy adult men, cephalexin achieved higher and more rapid maximum plasma concentrations, whereas cefadroxil demonstrated a longer elimination half‐life and higher area under the drug‐concentration time curve; both drugs demonstrated log‐linear elimination [29].

Higher doses of cephalexin and cefadroxil yield higher maximum plasma concentrations and area under the curves (AUCs), but the half‐life and time to peak concentrations are unchanged [29]. Among children weighing more than 70 kg (similar weights to adults) with MSSA isolates with an MIC distribution almost entirely ≤ 4 mg/L, cephalexin doses of 2250–4000 mg per day (divided in two to four doses) as well as cefadroxil doses of 3000–4500 mg per day (divided in two to three doses) achieved probability of target attainments of > 90% for 40% fT>MIC of organisms with MICs 1–4 mg/L [19]. These data are reassuring for BHS, which are the most common pathogens in nonpurulent cellulitis, where MIC values are lower. However, cefadroxil doses of 1000 mg two times daily and 1500 mg two times daily were not able to achieve PTAs > 90% for MICs of 4 mg/L. It is challenging to extrapolate from pediatric studies to adults even when evaluating older children who more closely approximate adult body weights. However, using worst‐case scenario simulations applied to adult patients versus MSSA isolates with MICs of 4 mg/L, higher doses of cephalexin (1 g three to four times daily) and cefadroxil (1–1.5 g three times daily or 2 g two times daily) are likely necessary to achieve a minimally adequate (> 40% fT>MIC) PTA. These authors suggest that for patients weighing more than 70 kg that cefadroxil doses of 1500–2000 mg two times daily be utilized. This study does not address whether lower doses of cephalexin are able to achieve similar PTAs but does show that at lower MIC thresholds PTAs are more likely to be achieved with cephalexin.

In adults, the United States Committee on Antimicrobial Susceptibility Testing (USCAST) has developed breakpoints for cephalexin for *S. aureus* utilizing PK/PD modeling from neutropenic thigh mouse models [30]. Based on their modeling, the MIC breakpoint for cephalexin against *S. aureus* was set at ≤ 8 mg/L. However, this was only for the cephalexin dose of 1000 mg four times daily, as the 500 mg four times daily dose's PTA was approximately 50% for an MIC of 8 mg/L, although PTA was approximately 90% for MIC of 4 mg/L. The other caveat from USCAST was that this breakpoint was based on a net bacterial stasis end point, so the recommendation was that this breakpoint only be used for non‐severe, uncomplicated infections. Among available information from USCAST, other cephalexin dosing strategies and cefadroxil dosing strategies were not performed.

Drug penetration and concentration into infected/inflamed soft tissues is also relevant in SSTI. A skin abrasion study in six healthy men found cephalexin levels in the skin were approximately 75% of those in serum [31]. In healthy adult skin blister models comparing cefadroxil to cephalexin, cefadroxil had numerically higher peak blister levels (20 μg/mL vs. 13 μg/mL, respectively), a higher proportion of blister fluid levels compared to serum, and longer excretion times from the blister fluid [32, 33]. A recent systematic review of cephalexin PK did not find any studies evaluating PK in patients with SSTIs [34]. Thus, limited data inform how cephalexin and cefadroxil PK/PD may be altered in patients with active SSTI or its common comorbidities (e.g., lymphedema). Additional studies evaluating cephalexin and cefadroxil PK/PD in medically complex and comorbid patients are desirable.

## 6 Clinical Data on Optimal Dosing and Frequency of Cephalexin

Limited, older data support the package labeling of two times daily cephalexin for SSTI [13, 16]. A 1981 study of 133 patients with SSTI (including 58% impetigo and only 14% cellulitis) compared cephalexin dosing of 500 mg two times daily with 250 mg four times daily and found similar efficacy (100% vs. 97% clinical cure, respectively) [16]. Also from 1981, a multicenter, randomized, double‐blind trial comparing cephalexin regimens of 500 mg two times daily to 1 g two times daily in 234 patients with SSTI found that favorable clinical response at day 6 was similar between groups: 85% for 500 mg two times daily versus 88% for 1 g two times daily [13]. A 1970s RCT compared 7 days of cephalexin 250 mg four times daily with 500 mg four times daily in 217 patients with SSTI, where a satisfactory response at end of therapy was achieved in 95% in the 250‐mg group compared to 92% in the 500‐mg group, suggesting lower dosing was adequate [12]. Although these results are encouraging for less frequent dosing of cephalexin, a lack of comparison with higher doses, inadequate statistical methodologies (i.e., lack of power calculations or confidence intervals), inclusion of mild SSTIs (i.e., impetigo), and potential differences in patient populations over time (e.g., obesity rates) limits applicability to modern moderate–severe SSTI infections.

Modern studies have used higher cephalexin doses; however, clinical outcomes and adverse events have not been stratified by dosing regimen or weight. A recent pilot open‐label RCT from Australia (*n* = 47) compared oral cephalexin 1 g four times daily to intravenous cefazolin 2 g two times daily and found oral cephalexin was non‐inferior for mean days to no cellulitis advancement, with a non‐significant trend toward higher treatment failure in the intravenous cefazolin group (4% with cephalexin vs. 22% with cefazolin, *p* = 0.10) [35].

Two studies on cellulitis did not identify a relationship between treatment failure and cephalexin dosing [36, 37, 38]. In a retrospective study of 48 outpatients with uncomplicated cellulitis who received cephalexin, 19 patients had therapeutic failure (composite of increase in antibiotic dose, extended duration, substitution for different antibiotic, or return visit requiring surgery or hospitalization), whereas 29 patients had therapeutic nonfailure; there was no significant difference in the mean daily dose between groups (1.73 g for therapeutic failure group vs. 1.57 g for nonfailure group, *p* = 0.23) [36]. In a post hoc analysis of a multicenter observational cohort study of 359 outpatients with uncomplicated cellulitis who received cephalexin, 69% of patients received 500 mg four times daily while 10.3% received higher doses; dose‐related factors were not predictive of clinical failure (prolonged antibiotic duration, addition/substitution of initial antibiotic, return visit, or incision and drainage) [37, 38]. However, the absence of an observed difference in these studies is limited by selection bias and small sample sizes.

Most recently, a pilot RCT evaluating cellulitis in Canada compared cephalexin 1000 mg four times daily (*n* = 33) to 500 mg four times daily (*n* = 33) for 7 days [15]. Despite higher proportions of obesity (54.5% vs. 36.4%) and diabetes mellitus (24.2% vs. 6.1%) in the high versus standard dose arms, oral antibiotic treatment failure was numerically higher in the standard (12.9%) versus high‐dose arm (3.2%). However, overall clinical cure at day 14 was similar (45.2% with high dose vs. 38.7% with standard), and a concomitant numerical increase in adverse events with higher dosing—primarily nausea/vomiting and diarrhea—was observed (38.7% with high dose vs. 25.8% with standard). As this trial was exploratory in nature and powered only for feasibility, no statistical comparisons were performed for these outcomes. Given these results suggest a potential for clinical benefit with high‐dose cephalexin (albeit at the expense of gastrointestinal tolerability), the same investigators are recruiting for an estimated 446‐participant double‐blind RCT (NCT05852262) with similar interventions and primary outcomes as the pilot RCT, which has been termed HI‐DOCC (HIgh‐DOse Cephalexin for Cellulitis) with an estimated completion in 2026 [39].

## 7 Clinical Data on Optimal Dosing and Frequency of Cefadroxil

Limited data also support less frequent cefadroxil dosing for SSTI. In 1976, a single author reported treatment of 36 consecutive patients with SSTI with either cefadroxil 300 mg three times daily (*n* = 25) or 300 mg two times daily (*n* = 11), achieving clinical and microbiologic cures in all patients; although this small observational study is subject to confounding by indication [17]. In 1982, a double‐blind trial compared cefadroxil 1000 mg daily (*n* = 60), cefadroxil 500 mg two times daily (*n* = 59), and cephalexin 500 mg four times daily (*n* = 61) in patients with SSTI [14]. The overall clinical and bacteriologic effectiveness were 98%, 97%, and 98%, respectively. Although these results are encouraging given the high efficacy rates (greater than in current studies), this publication lacks the characteristics requisite for meaningful interpretation, including patient characteristics, sound methodology (e.g., statistical comparisons and power analysis), and adverse event profiles [14].

In the same 1982 publication, a series of open‐label and blinded trials compared cephalexin and cefadroxil dosing regimens for clinical effectiveness in SSTI [14]. In an open‐label study of 373 patients, a combined outcome of clinically and bacteriologically satisfactory response was 99% versus 93% between cefadroxil 500 mg two times daily and 1000 mg two times daily, respectively [14]. This publication also compared cefadroxil 500 mg two times daily (*n* = 18), cefadroxil 1000 mg two times daily (*n* = 18), and cephalexin 500 mg two times daily (*n* = 22) in patients with furunculosis, finding an overall clinical and bacteriologic effectiveness of 100%, 100%, and 91%, respectively (cephadrine was also compared in this study) [14]. These results should be interpreted cautiously given the focus on furunculosis (a fairly mild infection for which antibiotics are often unnecessary) and limited reporting of patient characteristics, adverse events, and overall methodologic rigor that falls short of modern standards for comparative studies [14].

A RCT compared cephalexin 500 mg two times daily to cefadroxil 500 mg two times daily (additionally the study had a cefuroxime arm), each administered for 10 days for patients with SSTI [18]. At the end of therapy, cephalexin and cefadroxil produced similar rates of satisfactory clinical response (80/90 [88.9%] vs. 82/87 [94.3%], *p* = 0.281) and satisfactory microbiologic response (60/71 [84.5%] vs. 63/68 [92.6%], *p* = 0.185), respectively. Recurrences were numerically, but not significantly, higher with cephalexin versus cefadroxil (9/90 [10%] vs. 3/87 [3.4%]).

## 8 Are Higher Doses of Cephalexin and Cefadroxil Necessary in Obesity?

Obesity complicates up to 50% of SSTI cases and is a risk factor for SSTI development, treatment failure, and hospital readmission [2, 40, 41]. Obesity affects the PK/PD of many antimicrobials, not only increasing Vd, which may extend a drug's half‐life, but also increasing renal clearance, which may shorten the half‐life [42, 43]. The net impact of obesity on cephalexin and cefadroxil dosing is uncertain and remains inadequately studied. A comprehensive review on antibiotic dosing in obese adults suggests higher dosing of cephalexin (500–1000 mg four times daily) for severe or deep‐seated infections but does not cite references to support this recommendation [42]. Meanwhile, no recommendations for cefadroxil dosing are offered.

A conference abstract comparing PK/PD profiles of cephalexin in 19 hospitalized patients with and without obesity found no difference regarding systemic clearance or Vd [44]. One retrospective study found similar rates of therapeutic failures with cephalexin in cellulitis between patients with body mass index (BMI) < 30 kg/m^2^ versus ≥ 40 kg/m^2^ (10/69 [14.5%] vs. 5/25 [20%], *p* = 0.53) [45]. The majority (83/94 [88.3%]) of patients were prescribed cephalexin 500 mg four times daily rather than two or three times daily dosing, and no comparisons were made between differing dosing strategies within these populations. Unfortunately, patients with obesity are poorly represented in SSTI RCTs with only 29/69 (42%) of RCTs recording weight and/or body mass index, including only one of five RCTs that included cephalexin or cefadroxil identified [46]. None of these trials stratified outcomes based on weight; thus, the optimal dosing of cephalexin and cefadroxil for SSTI in obesity remains poorly characterized.

## 9 First‐Generation Cephalosporin PK/PD From Population Model‐Based Simulations

We identified a single well‐conducted population PK study of cephalexin and cefadroxil in pediatric patients [19]. The model parameters for both drugs were allometrically scaled to adult body weight (70 kg). The population mean estimates utilized in our simulations were similar to PK estimates from prior studies, indicating that our simulations perform well with this allometric scaling factor [34, 47]. Thus, the population model from Haynes et al. was considered fit for the purpose of simulating exposures in adults [19].

**FIGURE 1:**
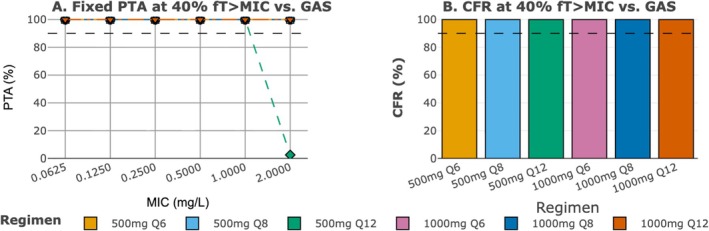
Probability of target attainment simulation (A) and cumulative fraction of response (B) for cephalexin versus typical *Streptococcus pyogenes* MIC ranges at 40% fT>MIC. (A) PTA of dosing regimens of cephalexin for a goal of > 40% fractional time above MIC for typical *S. pyogenes* MICs. (B) CFR of dosing regimens of cephalexin for a goal of > 40% fractional time above MIC for typical *S. pyogenes* MICs. 15% protein binding was used for all simulations. CFR, Cumulative Fraction of Response; GAS, Group A *Streptococcus*; MIC, Minimum inhibitory concentration; PTA, Probability of target attainment.

**FIGURE 2:**
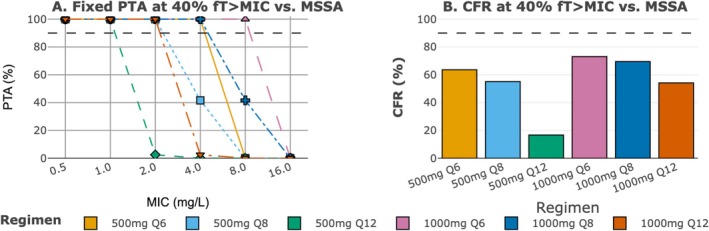
Probability of target attainment simulation (A) and cumulative fraction of response (B) for cephalexin versus typical methicillin‐susceptible *Staphylococcus aureus* MIC ranges at 40% fT>MIC. (A) PTA of dosing regimens of cephalexin for a goal of > 40% fractional time above MIC for typical methicillin‐susceptible *Staphylococcus aureus* MICs. (B) CFR of dosing regimens of cephalexin for a goal of > 40% fractional time above MIC for typical methicillin‐susceptible *Staphylococcus aureus* MICs. 15% protein binding was used for all simulations. CFR, Cumulative Fraction of Response; MSSA, Methicillin‐susceptible *Staphylococcus aureus* ; MIC, Minimum inhibitory concentration; PTA, Probability of target attainment.

**FIGURE 3:**
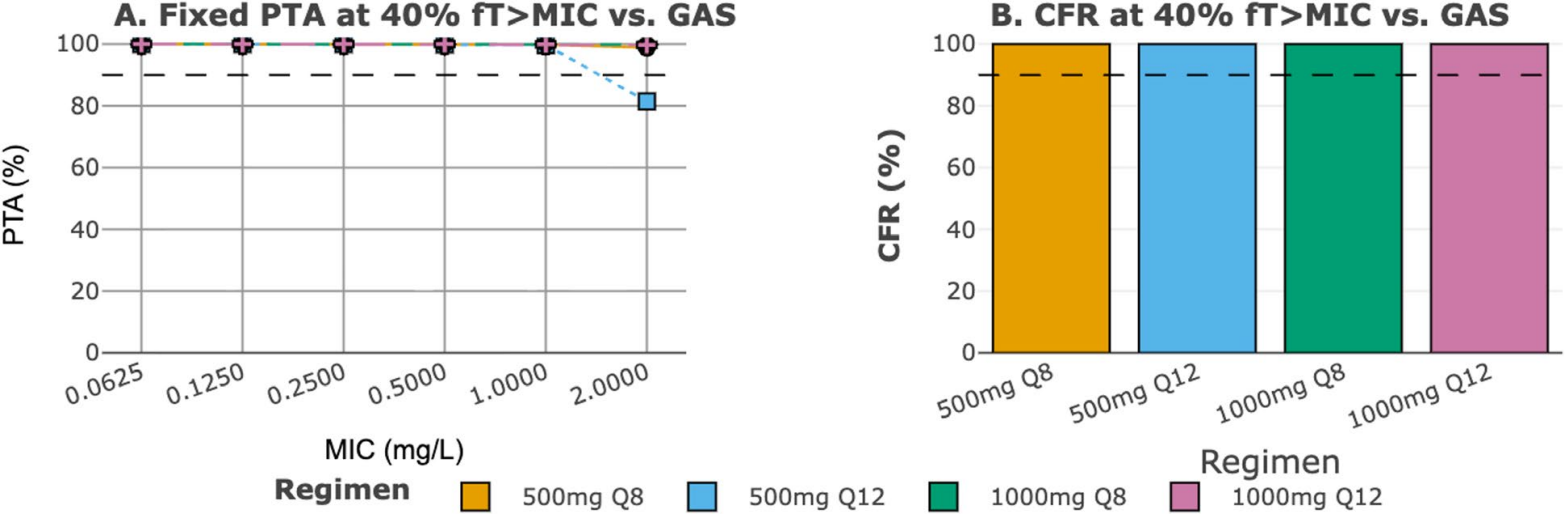
Probability of target attainment simulation (A) and cumulative fraction of response (B) for cefadroxil versus typical *Streptococcus pyogenes* MIC ranges at 40% fT>MIC. (A) PTA of dosing regimens of cefadroxil for a goal of > 40% fractional time above MIC for typical *S. pyogenes* MICs. (B) CFR of dosing regimens of cefadroxil for a goal of > 40% fractional time above MIC for typical *S. pyogenes* MICs. 15% protein binding was used for all simulations. A CFR threshold of 90%, represented as a dotted line, was used to define adequate population‐level target attainment. CFR, Cumulative Fraction of Response; GAS, Group A *Streptococcus*; MIC, Minimum inhibitory concentration; PTA, Probability of target attainment.

**FIGURE 4:**
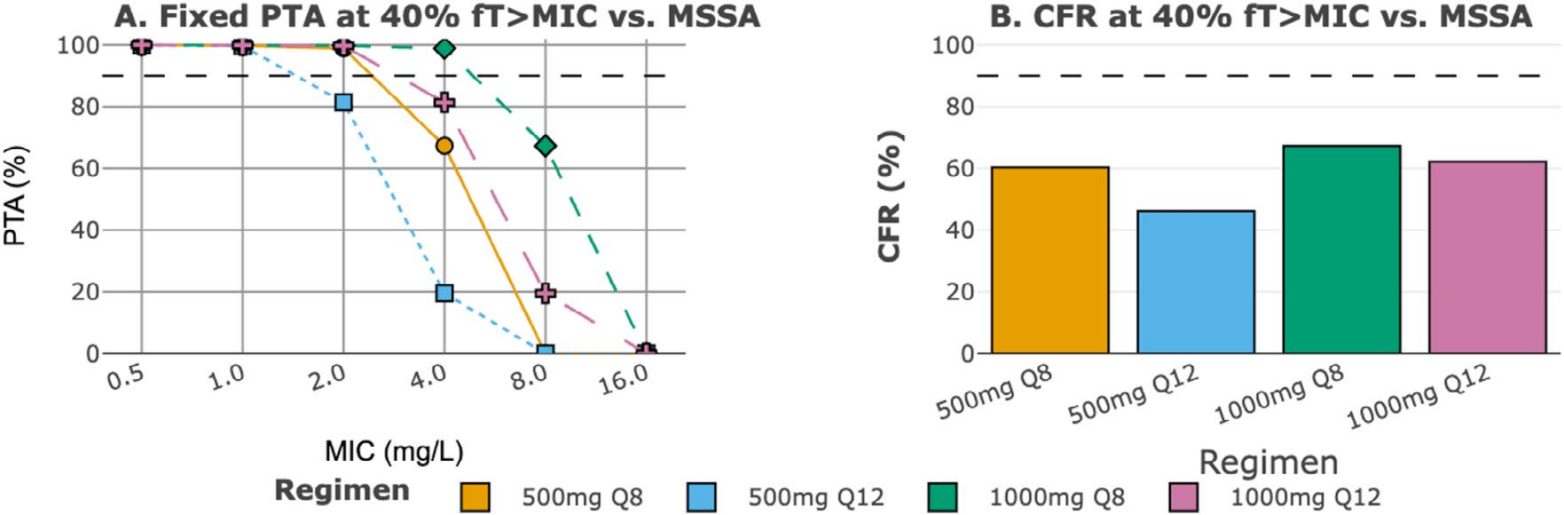
Probability of target attainment simulation (A) and cumulative fraction of response (B) for cefadroxil versus typical methicillin‐susceptible *Staphylococcus aureus* MIC ranges at 40% fT>MIC. (A) PTA of dosing regimens of cefadroxil for a goal of > 40% fractional time above MIC for typical methicillin‐susceptible *Staphylococcus aureus* MICs. (B) CFR of dosing regimens of cefadroxil for a goal of > 40% fractional time above MIC for typical methicillin‐susceptible *Staphylococcus aureus* MICs. 15% protein binding was used for all simulations. A CFR threshold of 90%, represented as a dotted line, was used to define adequate population‐level target attainment. CFR, Cumulative Fraction of Response; MSSA, Methicillin‐susceptible *Staphylococcus aureus* ; MIC, Minimum inhibitory concentration; PTA, Probability of target attainment.

The PTA and CFR analyses are summarized in Figures 1, 2, 3, 4 for GAS and MSSA, respectively, at a goal of 40% *f*T>MIC. For cephalexin treatment of GAS (Figure 1), all simulated dosing regimens achieved 100% CFR and > 90% PTA at all MICs for 40% *f*T>MIC except for 500 mg two times daily regimens at MICs ≥ 2 mg/L. For cephalexin treatment of MSSA (Figure 2), regimens of 1000 mg two times daily or > 500 mg three to four times daily provided > 90% PTA for a goal of 40% *f*T>MIC at MICs up to 2 mg/L, but for MICs of 4 mg/L, only 500 mg four times daily or 1 g three to four times daily were able to achieve > 90% PTA. None of the dosing regimens were able to achieve CFRs of 90% for MSSA, but cephalexin 500 mg four times daily and 1 g three to four times daily had CFRs > 60%. For cefadroxil treatment of GAS (Figure 3), all simulated dosing regimens achieved 100% CFR and > 90% PTA at all MICs evaluated for a goal of 40% *f*T>MIC except for 500 mg two times daily. For cefadroxil treatment of MSSA (Figure 4) at a goal of 40% *f*T>MIC, only 1000 mg three times daily achieved > 90% PTA at MICs up to 4 mg/L. For MICs up to 2 mg/L, all regimens were able to achieve > 90% PTA except for 500 mg two times daily. No cefadroxil dosing regimens achieved CFRs of 90% for MSSA, but all regimens were > 60% except cefadroxil 500 mg two times daily. In Figures S1–S4, PTA and CFR analyses are conducted for a goal of ≥ 90% *f*T>MIC, which serves as a conservative estimate and may be more applicable to severe infections.

## 10 Discussion

The PK/PD of cephalexin and cefadroxil in serum and soft tissue are described in healthy adults. However, there is a paucity of data describing these drugs' PK/PD in patients with obesity and active infection, and the clinical studies comparing dosing regimens for cephalexin and cefadroxil in SSTI are few, underpowered, largely in patients > 30 years old, primarily based on mild severity infections in outpatients, and suffer numerous methodologic limitations with underreporting of relevant data. In addition, the pathogen causing cellulitis is rarely known, although studies suggest the vast majority of cellulitis is due to BHS, whereas purulent infections are much more likely to be caused by *S. aureus* [7]. Although labeled and guideline‐recommended doses might be adequate in BHS SSTI infections, the optimal dose of cephalexin and cefadroxil in obesity or MSSA infections is less certain.

Higher doses of first‐generation cephalosporins are well tolerated, but it is unclear if they offer improved efficacy. Less frequent administration than the four times daily dosing of cephalexin is likely adequate for mild SSTI from BHS, with older studies reporting high efficacy with two times daily regimens. Cefadroxil's half‐life supports two times daily administration, with limited older data suggesting success in SSTI even with daily dosing. However, these less frequent and lower dosed regimens are likely inadequate for SSTIs caused by *S. aureus* . Notably, our simulation analyses are broadly similar to the analyses conducted by USCAST for cephalexin for *S. aureus* in that we found that only doses of 1000 mg four times daily achieve high PTA for MICs of 8 mg/L [30]. Our study adds additional modeling for GAS, for cefadroxil, and for additional less frequent dosing strategies.

Our simulation data suggest that the impact of the PK/PD differences between cefadroxil and cephalexin may be overstated. For both drugs, > 40% *f*T_>MIC_ for typical staphylococcal MICs up to 4 mg/L could only reliably be achieved with three times daily or more frequent dosing, whereas the more relaxed target of > 40% *f*T_>MIC_ for typical streptococcal MICs could be achieved with either drug dosed as infrequently as two times daily. Interestingly, cefadroxil dosed two times daily appeared less reliable than equivalent doses of cephalexin at achieving > 90% *f*T_>MIC_ for MICs 0.125–0.25 mg/L, whereas cefadroxil was slightly more active at a 1000 mg three times daily than the equivalent dose of cephalexin at an MIC of 1 mg/L. Regardless, these differences would be outweighed by even a two‐fold variation in cephalexin versus cefadroxil MIC for a given pathogen; older studies suggested relevant pathogens have two‐fold lower MICs to cefadroxil, whereas one modern cohort suggests MICs for the two agents are similar, and thankfully have not increased much over the past decades [48, 49, 50].

Modern RCTs, such as HI‐DOCC, should soon shed light on the optimal dosing of cephalexin for this common infection with high rates of clinical failure and morbidity, in particular whether higher doses than current IDSA guideline recommendations are warranted [10, 39]. Pragmatic RCTs would be welcome to compare cephalexin and cefadroxil for SSTI to determine whether achievement of similar clinical outcomes with less burdensome two times daily dosing regimens is possible. Additional pathogen‐focused PK/PD studies of these agents could assist with dose selection in challenging patient populations.

Our literature review and analysis has some limitations. This was not a formal systematic review, and the studies that were identified were older, methodologically flawed, and primarily in mild, outpatient infections. Our PK/PD simulations leveraged the variability observed in pediatric patients with parameter values extrapolated to adults, but it is likely that a wider range of adult patients with differing levels of renal dysfunction would potentially lead to different results. Although we utilized a pediatric model structure for our simulations and acknowledge that there may be higher Vd in adults compared to pediatrics, our adult simulation utilizing allometric scaling led to Vd consistent with the literature on cephalexin and cefadroxil in adults. We chose to utilize this PK/PD pediatric model as our base due to its robust nature and additional covariate information compared to previous PK/PD models with oral cephalosporins. Further, the pediatric PK model we utilized includes allometric scaling, which supports parameter scaling across body sizes. Rapid maturation is likely most relevant for ages < 1 year (not applicable to our simulations). Given the model was developed in pediatric patients aged 0.5–18 years (weight range 7.5–79 kg), the average parameter estimates (weight‐normalized) represent a reasonable starting point [19]. Additional PK/PD studies are needed to define optimal dosing for these agents, particularly in older adults with renal dysfunction. Our PK/PD analysis likely represents a worst‐case scenario for oral cephalosporins, as pediatric CL estimates approached those of healthy adult volunteers, and renal function was not explicitly evaluated in our simulations. Thus, patients with greater degrees of renal dysfunction may be at risk of drug accumulation and possible dose‐related side effects with aggressive dosing. Clinicians must weigh the trade‐offs of overdosing versus underdosing, as real‐time drug assays for these agents are not yet widely available. Our PK/PD modeling used plasma drug exposures, rather than tissue drug exposures, which could potentially overestimate the effectiveness of agents, especially in tissue abscesses, although using a target of ≥ 90% *f*T_>MIC_ provides conservative estimates for achieving appropriate target attainment. Further, with increasing emphasis on early intravenous‐to‐oral switch or full oral therapy for severe infections (e.g., bacteremia), a more aggressive initial dose (targeting ≥ 90% *f*T_>MIC_) also represents a more conservative approach [51, 52]. Regarding tissue penetration, it is notable that differences in plasma and tissue penetration can occur. However, beta‐lactams typically demonstrate adequate penetration into skin and soft tissue and free drug concentration in tissues approximately correlate to free drug plasma concentrations, especially in the setting of inflammation [53, 54, 55]. Additionally, SSTI is a heterogenous syndrome including impetigo to cutaneous abscess to necrotizing soft tissue infections with wide‐ranging severity. This heterogeneity may make interpreting and utilizing PK/PD modeling challenging to interpret the exact *f*T_>MIC_ target attainment needed for the various clinical syndromes evaluated herein, but may also affect interpretation of limited clinical data primarily of mild outpatient infections challenging to extrapolate to moderate–severe SSTIs that have received initial intravenous therapy or debridement. Limitations of the current simulations include lack of robust data linking PK/PD to clinical success for adults with SSTIs. As such, linking target attainment to outcomes is challenging in the absence of studies explicitly linking PK/PD target attainment to outcomes for SSTIs [30]. Lastly, our simulations do not account for any potential inoculum effect of *S. aureus* . However, it is important to note that prevalence of *blaZ* genes conferring the inoculum effect in MSSA isolates varies considerably throughout the world, and SSTIs with appropriate source control procedures are less likely to be impacted by any inoculum effect, in contrast to endovascular infections [56, 57].

## 11 Conclusions

Optimal dosing of cephalexin and cefadroxil for SSTIs warrants further exploration with modern trial and PK/PD methodology. Given potential risks of underdosing due to obesity, non‐adherence with frequently dosed regimens, and inadequate PTA in higher MIC organisms (e.g., MSSA), we favor using higher‐dosed regimens such as cephalexin 1000 mg three times daily or cefadroxil 1000 mg two times daily for moderate–severe SSTIs compared to current IDSA recommendations for cephalexin 500 mg four times daily. However, the clinical and PK/PD data to support one regimen over another or the exact dosing regimen remains incomplete. Lower and less frequent doses may remain efficacious, especially for mild, streptococcal SSTIs, which may improve patient tolerability and adherence, but this needs prospective confirmation.

## Funding

The authors have nothing to report.

## Conflicts of Interest

T.C.V.S. reports receiving consultant and research funding from bioMérieux and research funding from MannKind, which are not related to this work. J.H.R. reports travel support and speaking payments from bioMérieux, which are not related to this work. The other authors declare no conflicts of interest.

## Supporting information


**Table S1:** Studies Comparing Cephalexin (LEX) and Cefadroxil (CFR) Pharmacokinetics and Pharmacodynamics.
**Figure S1:** Probability of target attainment simulation (A) and cumulative fraction of response (B) for cephalexin versus typical Streptococcus pyogenes MIC ranges at 90% fT>MIC.
**Figure S2:** Probability of target attainment simulation (A) and cumulative fraction of response (B) for cephalexin versus typical methicillin‐susceptible Staphylococcus aureus MIC ranges at 90% fT>MIC.
**Figure S3:** Probability of target attainment simulation (A) and cumulative fraction of response (B) for cefadroxil versus typical Streptococcus pyogenes MIC ranges at 90% fT>MIC.
**Figure S4:** Probability of target attainment simulation (A) and cumulative fraction of response (B) for cefadroxil versus typical methicillin‐susceptible Staphylococcus aureus MIC ranges at 90% fT>MIC.

## Data Availability

The data that support the findings of this study are available from the corresponding author upon reasonable request.
