# Supplementary material for: Getting It Right the Second Time: How Can we Optimize First‐Generation Cephalosporin Dosing for Skin and Soft Tissue Infections in the 21st Century?
Source: Pharmacotherapy. 2026 Jun 10;46(7):e70179. doi: 10.1002/phar.70179 (PMC13254493; doi:10.1002/phar.70179)
Supplement: Supplementary file 1 — Table S1: Studies Comparing Cephalexin (LEX) and Cefadroxil (CFR) Pharmacokinetics and Pharmacodynamics. Figure S1: Probability of target attainment simulation (A) and cumulative fraction of response (B) for cephalexin versus typical Streptococcus pyogenes MIC ranges at 90% fT>MIC. Figure S2: Probability of target attainment simulation (A) and cumulative fraction of response (B) for cephalexin versus typical methicillin‐susceptible Staphylococcus aureus MIC ranges at 90% fT>MIC. Figure S3: Probability of target attainment simulation (A) and cumulative fraction of response (B) for cefadroxil versus typical Streptococcus pyogenes MIC ranges at 90% fT>MIC. Figure S4: Probability of target attainment simulation (A) and cumulative fraction of response (B) for cefadroxil versus typical methicillin‐susceptible Staphylococcus aureus MIC ranges at 90% fT>MIC. [file PHAR-46-0-s001.docx]

**Supplemental Materials**

**Supplemental Methods**

Search Strategy

We searched PubMed with the query “(cephalexin[title] OR cefadroxil[title] OR "oral beta-lactam"[title] OR "oral cephalosporin"[title] OR "first-generation cephalosporin"[title]) AND (dose[title/abstract] OR dosing[title/abstract] OR dosage[title/abstract] OR pharmacokinetics[title/abstract] OR pharmacodynamics[title/abstract] OR high-dose[title/abstract] OR obesity[title/abstract] OR weight-based[title/abstract] OR "randomized controlled trial"[Publication Type] OR "clinical trial"[Publication Type] OR pharmacology[title/abstract]).” The search was done on January 23, 2025 and identified 376 English-language articles.

Pharmacokinetic simulation methods

Population pharmacokinetic (PK) models were embedded into the Pmetrics package (v.2.0.0). for R (v.4.3.1) where we simulated cephalexin and cefadroxil target attainment. For each drug, mean parameter values were embedded along with inter-individual variability (ω^2^ = ln[(CV%/100)^2^ +1]) estimates as the diagonal of the variance-covariance matrix.[1] Covariance (i.e., the off diagonal of the matrix) was set equal to zero. Monte Carlo sampling was conducted from this unimodal distribution generating 1,000 parameter sets (i.e., simulated subjects) for each drug and dosing regimen. A free, unbound, fraction of 85% (fu = 0.85) was assumed for both drugs. We simulated cephalexin doses of 500 or 1000 mg orally every 6, 8, or 12 hours. For cefadroxil, we simulated doses of 500 or 1000 mg orally every 8 or 12 hours. We compared PK/pharmacodynamic (PD) target attainment versus minimum inhibitory concentrations (MICs) in doubling dilutions for Group A *Streptococcus* (GAS) (0.06 to 0.5 mg/L) and methicillin-susceptible *Staphylococcus aureus* (MSSA) (0.5 to 16 mg/L). PK/PD target attainment was evaluated versus goals of 40% or 90% free time above MIC (*f*T_>MIC_) for each regimen, based on prior studies.[2, 3]

Cumulative fraction of response (CFR)

A CFR analysis was performed to estimate regimen-level target attainment across organism-specific MIC distributions. MIC distributions for each organism were taken from European Committee on Antimicrobial Susceptibility Testing when available. When unavailable, cefuroxime MIC distributions were used as a proxy comparator. MIC values and isolate counts were converted into target distributions, and CFR was calculated using simulated concentration-time profiles with target type specified as time above MIC over the 0–24 h dosing interval. A free fraction of 0.85 was applied. CFR was evaluated at predefined PK/PD targets of 40% and 90% free time above MIC. Results were expressed as the percentage of simulated subjects achieving the target across the weighted MIC distribution for each organism-drug pair. A CFR threshold of 90%, represented as a dotted line in each figure, was used to define adequate population-level target attainment.

**Supplemental Table: Studies Comparing Cephalexin (LEX) and Cefadroxil (CFR) Pharmacokinetics and Pharmacodynamics**

|  | **Pfeffer (1977)[4]** | **Hartstein (1977)[5]** | **Lode (1979)[6]** | **Simon (1980)[7]** | **Welling (1985)[8]** | **Barbhaiya (1996)[9]** |
| --- | --- | --- | --- | --- | --- | --- |
| Patient Population | N=12  Sex: 11 male, 1 female  Age (range): 23-59 years  Weight (range): 51-83 kg | N=20  Sex: 20 male  Age (range): 23-44 years  Weight (range): 63-100 kg; (mean) 79 kg | N=12  Sex: 6 male, 6 female  Age (range): 20-41 years  Weight (range): 52.5-76 kg; (mean) 67.8 kg | N=10  Sex: 10 male  Age (range): 25-45 years  Weight: NR | N=12  Sex: 12 male  Age (mean): 24 years  Weight (range): 70-91 kg; (mean) 80 kg | N=36  Sex: 36 male  Age (mean): 27.2 years  Weight (mean): 69.9 kg |
| Drugs and Dosages | Cephalexin 500 mg  Cefadroxil 500 mg  Cephradine 500 mg* | Cephalexin 50 0mg  Cefadroxil 500 mg, 100 mg | Cephalexin 1000 mg  Cefadroxil 1000 mg  Cefaclor*1000 mg  Cefroxadine* 1000 mg | Cephalexin 1000 mg  Cefadroxil 1000 mg | Cephalexin 50 0mg  Cefadroxil 500 mg | Cephalexin 250 mg, 500 mg, 1000 mg  Cefadroxil 250 mg, 500 mg, 1000 mg |
| *C*_max_ (µg/ml) | LEX 500: 20.7  CFR 500: 16.2 | NR | LEX 1000: 38.8  CFR 1000: 33 | LEX 1000: 23.3  CFR 1000: 28.4 | LEX 500: 17.5  CFR 500: 16 | LEX 250: 12  LEX 500: 20.7  LEX 1000: 42  CFR 250: 8.7  CFR 500: 15.1  CFR 1000: 30.8 |
| *t*_max_ (hours) | LEX 500: 0.71  CFR 500: 1.29 | LEX 500: 1  CFR 500: 1.5  CFR 1000: 1.5 | LEX 1000: 0.93  CFR 1000: 1.71 | LEX 1000: NR  CFR 1000: NR | LEX 500: 1.02  CFR 500: 1.8 | LEX 250: 0.6  LEX 500: 0.6  LEX 1000: 0.6  CFR 250: 0.8  CFR 500: 1.3  CFR 1000: 0.9 |
| AUC (µgh/ml) | LEX 500: 29.0  CFR 500: 47.4 | NR | LEX 1000: 93  CFR 1000: 108.5 | LEX 1000: 60.0  CFR 1000: 95.9 | LEX 500: 24.9  CFR 500: 40.8 | LEX 250: 14.1  LEX 500: 29  LEX 1000: 59.9  CFR 250: 22.6  CFR 500: 44.8  CFR 1000: 93.1 |
| *t*_1/2_ (hours) | LEX 500: 0.57  CFR 500: 1.27 | NR | LEX 1000: 1.04  CFR 1000: 1.63 | LEX 1000: 1.2  CFR 1000: 1.5 | LEX 500: 0.68  CFR 500: 1.1 | LEX 250: 1.1  LEX 500: 1.0  LEX 1000: 1.1  CFR 250: 1.9  CFR 500: 2.1  CFR 1000: 2.1 |

*Results from drugs other than cefadroxil and cephalexin were not reported in this table

Abbreviations: AUC: area under the serum concentration vs time curve; CFR: cefadroxil; *C*_max_: maximum serum concentration; LEX: cephalexin; NR: not reported; *t*_1/2_: half-life; *t*_max_: time to peak serum concentration

**Supplemental Figures**

**Supplemental Figure 1: Probability of target attainment simulation (A) and cumulative fraction of response (B) for cephalexin versus typical *Streptococcus pyogenes* MIC ranges at 90% fT>MIC**

**
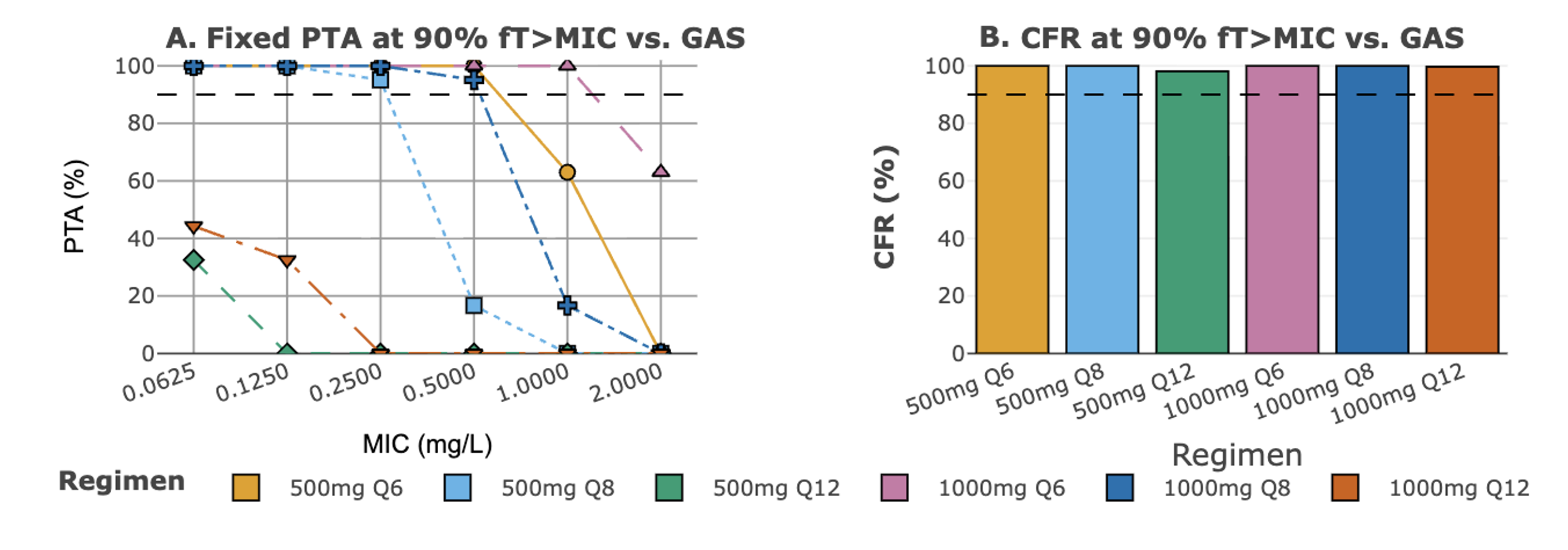
**

A: PTA of dosing regimens of cephalexin for a goal of >90% fractional time above MIC for typical *S. pyogenes* MICs. B: CFR of dosing regimens of cephalexin for a goal of >90% fractional time above MIC for typical *S. pyogenes* MICs. Note: 15% protein binding was used for all simulations. A CFR threshold of 90%, represented as a dotted line, was used to define adequate population-level target attainment. Abbreviations: CFR: cumulative fraction of response; GAS: Group A *Streptococcus*; MIC: minimum inhibitory concentration; PTA: probability of target attainment.

**Supplemental Figure 2: Probability of target attainment simulation (A) and cumulative fraction of response (B) for cephalexin versus typical methicillin-susceptible *Staphylococcus aureus* MIC ranges at 90% fT>MIC**


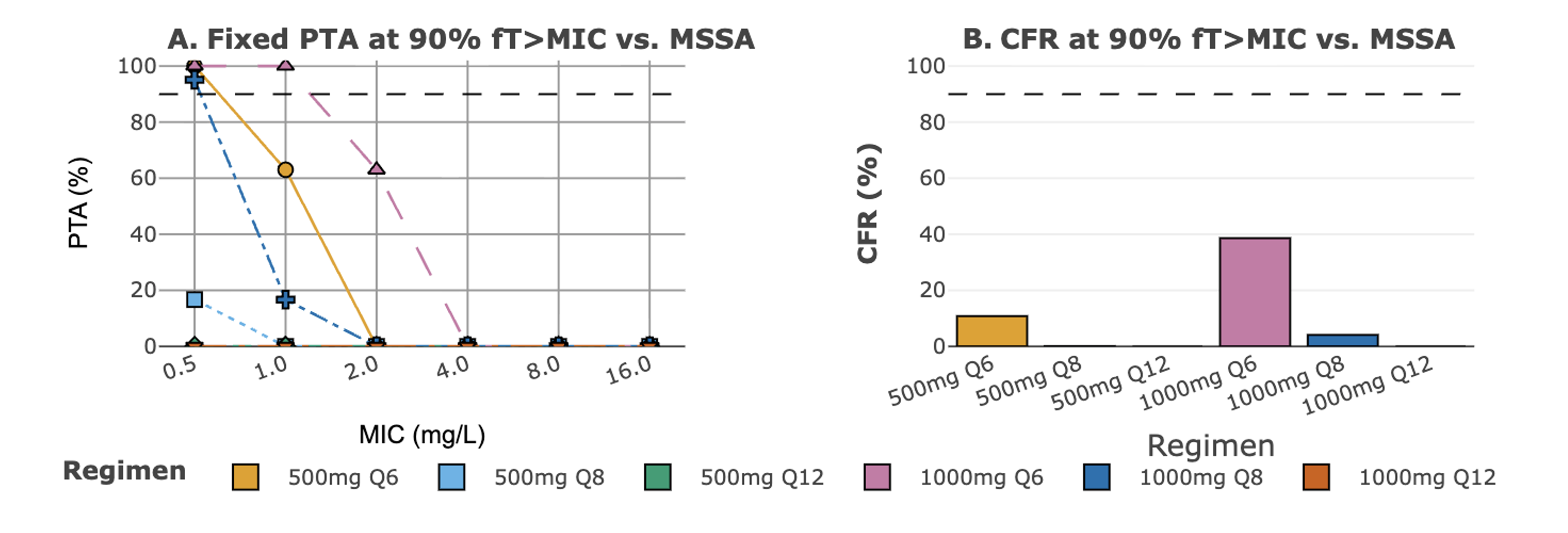


A: PTA of dosing regimens of cephalexin for a goal of >90% fractional time above MIC for typical methicillin-susceptible *Staphylococcus aureus* MICs. B: CFR of dosing regimens of cephalexin for a goal of >90% fractional time above MIC for typical *S. aureus* MICs. Note: 15% protein binding was used for all simulations. A CFR threshold of 90%, represented as a dotted line, was used to define adequate population-level target attainment. Abbreviations: CFR: cumulative fraction of response ; MIC: minimum inhibitory concentration; MSSA: methicillin-susceptible *Staphylococcus aureus*; PTA: probability of target attainment.

**Supplemental Figure 3: Probability of target attainment simulation (A) and cumulative fraction of response (B) for cefadroxil versus typical *Streptococcus pyogenes* MIC ranges at 90% fT>MIC**


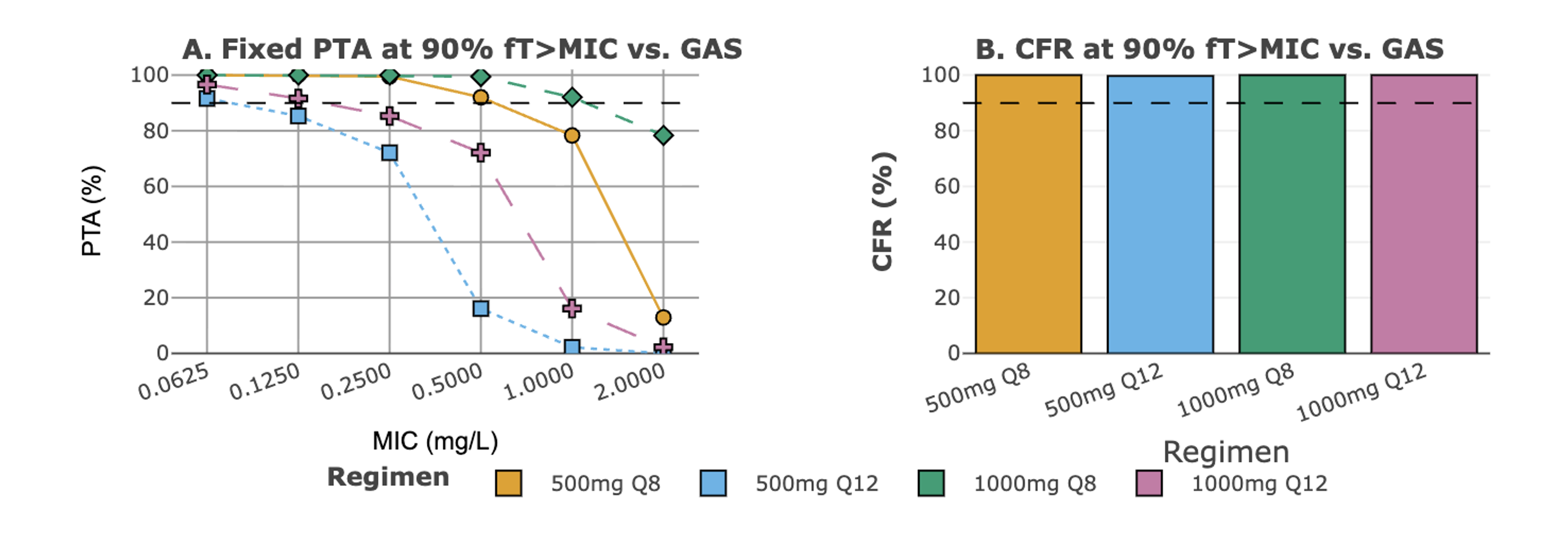


A: PTA of dosing regimens of cefadroxil for a goal of >90% fractional time above MIC for typical *S. pyogenes* MICs. B: CFR of dosing regimens of cefadroxil for a goal of >90% fractional time above MIC for typical *S. pyogenes* MICs. Note: 15% protein binding was used for all simulations. Abbreviations: CFR: cumulative fraction of response; GAS: Group A *Streptococcus*; MIC: minimum inhibitory concentration; PTA: probability of target attainment.

**Supplemental Figure 4: Probability of target attainment simulation (A) and cumulative fraction of response (B) for cefadroxil versus typical methicillin-susceptible *Staphylococcus aureus* MIC ranges at 90% fT>MIC**


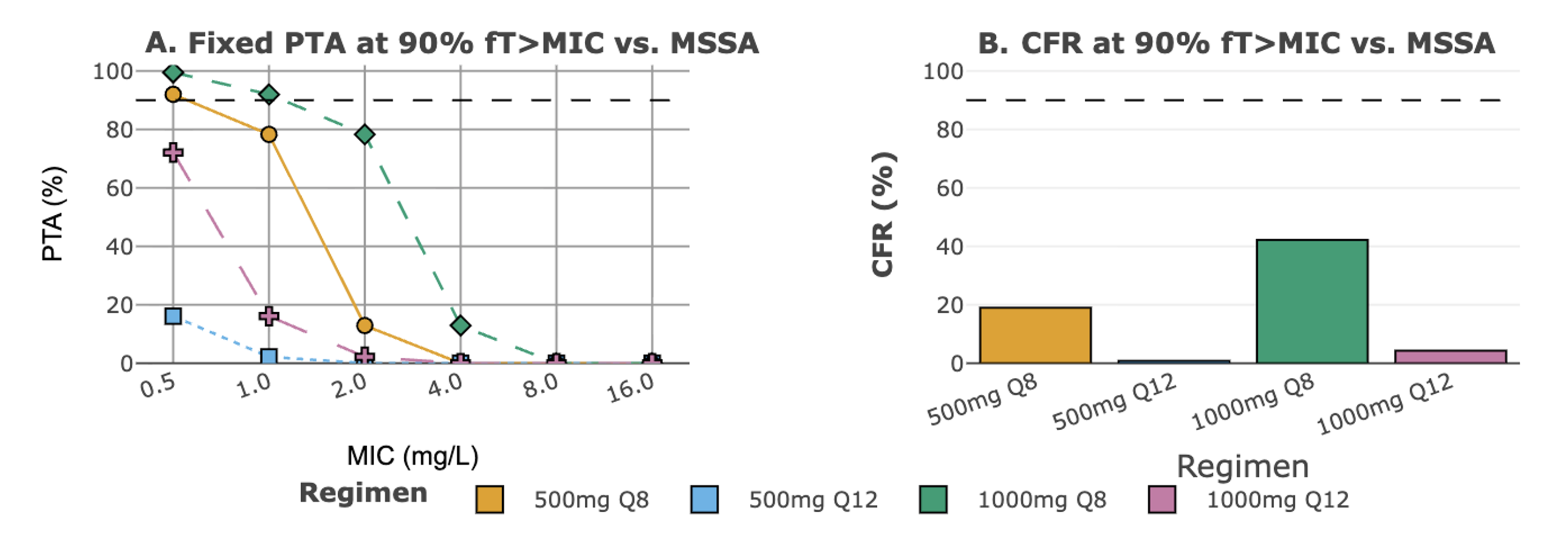


A: PTA of dosing regimens of cefadroxil for a goal of >90% fractional time above MIC for typical methicillin-susceptible *Staphylococcus aureus* MICs. B: CFR of dosing regimens of cefadroxil for a goal of >90% fractional time above MIC for typical *S. aureus* MICs. Note: 15% protein binding was used for all simulations. Abbreviations: CFR: cumulative fraction of response; MIC: minimum inhibitory concentration; MSSA: methicillin-susceptible *Staphylococcus aureus*; PTA: probability of target attainment.

**References**

1. Elassaiss-Schaap J, Heisterkamp S. Variability as constant coefficient of variation: Can we right two decades in error? Available at: <https://www.page-meeting.org/?abstract=1508>. Accessed January 21, 2025.

2. Craig WA, Ebert SC. Killing and regrowth of bacteria in vitro: a review. Scand J Infect Dis Suppl **1990**; 74: 63-70.

3. Scharf C, Liebchen U, Paal M, et al. The higher the better? Defining the optimal beta-lactam target for critically ill patients to reach infection resolution and improve outcome. J Intensive Care **2020**; 8(1): 86.

4. Pfeffer M, Jackson A, Ximenes J, de Menezes JP. Comparative human oral clinical pharmacology of cefadroxil, cephalexin, and cephradine. Antimicrob Agents Chemother **1977**; 11(2): 331-8.

5. Hartstein AI, Patrick KE, Jones SR, Miller MJ, Bryant RE. Comparison of pharmacological and antimicrobial properties of cefadroxil and cephalexin. Antimicrob Agents Chemother **1977**; 12(1): 93-7.

6. Lode H, Stahlmann R, Koeppe P. Comparative pharmacokinetics of cephalexin, cefaclor, cefadroxil, and CGP 9000. Antimicrob Agents Chemother **1979**; 16(1): 1-6.

7. Simon C. Zur Pharmakokinetik von Cefadroxil, einem neuen Oralcephalosporin. Infection **1980**; 8(5): S584-S7.

8. Welling PG, Selen A, Pearson JG, et al. A pharmacokinetic comparison of cephalexin and cefadroxil using HPLC assay procedures. Biopharm Drug Dispos **1985**; 6(2): 147-57.

9. Barbhaiya RH. A pharmacokinetic comparison of cefadroxil and cephalexin after administration of 250, 500 and 1000 mg solution doses. Biopharm Drug Dispos **1996**; 17(4): 319-30.
